# The lived experience of Iranian adults from coronavirus disease 2019 (COVID-19)—A qualitative study

**DOI:** 10.3389/fpubh.2024.1362708

**Published:** 2025-01-28

**Authors:** Samira Pourmoradian, Arezoo Haghighian-Roudsari, Tahereh Alsadat Khoubbin Khoshnazar, Ali Milani-Bonab

**Affiliations:** ^1^Nutrition Research Center, Department of Community Nutrition, Faculty of Nutrition and Food Sciences, Tabriz University of Medical Sciences, Tabriz, Iran; ^2^Department of Community Nutrition, Faculty of Nutrition Sciences and Food Technology, National Nutrition and Food Technology Research Institute, Shahid Beheshti University of Medical Sciences, Tehran, Iran; ^3^Nursing Care Research Center, School of Nursing and Midwifery, Iran University of Medical Sciences, Tehran, Iran; ^4^Food and Nutrition Policy and Planning Research Department, National Nutrition and Food Technology Research Institute and Faculty of Nutrition Sciences and Food Technology, Shahid Beheshti University of Medical Sciences, Tehran, Iran

**Keywords:** qualitative study, COVID-19, Iran, lived (passed) experience, Tehran (city)

## Abstract

**Introduction:**

The widespread impact of the coronavirus disease 2019 (COVID-19) epidemic on human health causes sudden lifestyle changes, through social distancing and isolation at home, with social and economic consequences. This qualitative study aimed to identify the lived experiences of recovered adult patients from COVID-19 in Iran.

**Methods:**

This qualitative research was conducted using a national phenomenological approach. The participants were patients who recovered from COVID-19 through their treatment period in the hospital or at home. Semi-structured in-depth interviews were applied for 45 participants who were invited using purposeful sampling and continued to achieve data saturation. The five-stage inductive process to analyze the structure of lived experience (IPSE) approach was used to analyze the data using MAXQDA 2020 software.

**Results:**

According to the statements of the participants in the study, five types of experiences extracted during the period of suffering from the disease: nutritional problems, physical problems, the psychological burden caused by the disease, the supporting role of others in the disease tolerance, and the unpleasant and better experiences of the disease.

**Discussion:**

Patients with COVID-19 disease experience many physical and psychosocial consequences which affect their quality of life. Therefore, sociopsychological support provided by psychologists and family members can have ameliorating effects in reducing disease consequences. Further interventional studies were needed to capture these aspects of diseases.

## Introduction

WHO announced coronavirus disease 2019 (COVID-19) as an epidemic disease in February 2020 (1). Therefore, the widespread impact on human health causes sudden lifestyle changes through social distancing and isolation at home, with social and economic consequences. Worldwide, more than 96.2 million people have been diagnosed with (COVID-19) disease, spreading rapidly worldwide (2). In 2020, more than 37% of COVID-19 patients were hospitalized in the UK, requiring invasive mechanical ventilation, with a mortality rate of more than 26% (3). A randomized trial in hospitalized, high-risk patients showed that nutritional support during hospitalization improves clinical outcomes, including survival (4). Nutritional deficiencies increase the risk of severe infection; for example, anorexia is one of the most common complications that patients experience during hospitalization and even 2 weeks after discharge (5). One of the factors affecting the rate of recovery and elimination of the disease is the severity of the infectious disease; so, in patients with mild-to-moderate types of COVID-19, it disappears faster in the recovery phase (6, 7). Social isolation was one of the solutions proposed by the health systems of many countries to deal with the spread of the disease, which caused changes in people's lifestyles (8). Factors such as the fear of being infected, contradictory knowledge about the factors affecting the spread of disease and the risk of death, minimal physical contact with friends and family, and the decrease in household income were among the things that threatened the lives of communities (9).

Iran was one of the countries that experienced many cases of this disease, and in different time intervals, it went through several high peaks of disease prevalence. In Iran, 7,533,087 people have been infected with COVID-19, fortunately, 7,307,292 patients have recovered or been discharged from hospitals (10). The COVID-19 pandemic prompted widespread health recommendations for home quarantine, significantly reducing individual, familial, and social interactions. Concerns regarding the transmission of the virus and its potentially fatal outcomes led to pervasive fear, stress, confusion, and hopelessness throughout Iranian society. Lifestyle changes and job insecurity exacerbated these psychological impacts. Additionally, the closure of public sports facilities and the apprehension toward public spaces, such as parks, increased physical inactivity and a noticeable decline in overall societal vitality (11). The results of a study in Iran showed that people had experiences such as death anxiety, the experience of stigma, the experience of ambiguity, positive emotional experiences, emotions experienced about family members, and the feelings caused by quarantine when suffering from a disease, which may have long-term adverse effects on people's physical and mental health (12).

In Iran, a significant number of individuals diagnosed with COVID-19 have effectively mitigated the overall severity of their condition throughout the illness and during the recovery phase by employing a strategic array of targeted behaviors and coping mechanisms. These approaches have collectively contributed to enhanced relaxation and an improved capacity to manage the challenges of the disease. Notable strategies include the cultivation of spiritual practices, the acquisition of knowledge regarding COVID-19, engagement in both meaningful and enjoyable activities, active participation in treatment protocols, fortification of inner spirit and fostering of hope, efforts to rectify previous errors, and the utilization of virtual communication to maintain social connections (13).

According to the prediction of the World Health Organization, this disease will become the third cause of death in the world by 2030. The specific complexity of this disease and its multiple dimensions and consequences play a role in creating significant health, social, and economic costs for individuals and societies (14). Qualitative research methods are particularly well-suited for exploring topics of significant complexity and numerous unknowns. By conducting interviews with individuals who have directly experienced the condition, researchers can uncover critical insights that may not be fully captured through quantitative approaches (15). Therefore, the present study aimed to identify the lived experiences of Iranian adults from COVID-19 related problems during the disease period to recognize the most crucial issues and offer solutions using qualitative design.

## Methods

### Study design

This qualitative research was conducted using a national interpretive phenomenological approach (16). The inductive process to analyze the structure of lived experience (IPSE) (17) was used to explore the lived experience of adults with COVID-19 in the Tehran (the capital of Iran) metro police.

This approach relies on an inductive process to explore the participants' lived experiences and deeply analyze their underlying concepts. Individuals express their actions, experiences, and feelings to the researcher who interprets their statements. This research method can provide information about the experiences and views of the research community to researchers. Conducting the study based on this method included the following steps.

#### Stage 1: setting up a research group

First, group members with diverse knowledge and backgrounds were recruited to facilitate the study's discoveries and novelty. The research group consisted of five members (three nutritionists, one nurse, and one medical doctor as team advisor), and among the research team members, four principal investigators had good experience designing and implementing qualitative studies.

#### Stage 2: ensuring the originality of the study

A member of the research group systematically reviewed qualitative and quantitative literature to review related and similar studies and verified that no study was currently being conducted on the experience of clinical practice during a pandemic in Iran. The other group members had access to this review only after completing the data analysis to ensure the inductive process and emphasize the study's novelty.

#### Stage 3: participants' recruitment and sampling

Two purposeful and convenience sampling strategies were used to select the participants in this study (18). Participants were recruited according to the inclusion and exclusion criteria outlined in Table 1. Purposive sampling was employed to select individuals who had recovered from COVID-19 and had been hospitalized due to the illness. Following a phone call to confirm their eligibility, they were invited to participate in the study. This sampling strategy aimed to include participants who could provide valuable insights and contribute new perspectives to the existing findings.

**Table 1:** Inclusion and exclusion criteria.

**Inclusion criteria**	**Exclusion criteria**
- Age ≥ 18 years - Person who has experienced COVID-19 - Agreed to participate in the study - Fluent in Persian	- Acute psychiatric and/or somatic symptoms hindering the conduct of a research interview such as hoarseness or lethargy

Another part of the participants was not among those referred to the hospitals; they were selected among those infected with COVID-19 and recovered using the convenience sampling method. This process continued until the saturation stage regarding safe data and themes was reached (19, 20).

#### Stage 4: data collection, access to experience

Permission was obtained to use the patients' information in coordination with the hospital's medical records department. Then, names and phone numbers of patients referred to the hospital's clinic due to COVID-19 were received. Researchers virtually conducted interviews using various methods, such as phone, email, and face-to-face (21, 22) interviews. The number of face-to-face interviews was very low due to the fear of disease transmission, and the majority of the participants preferred to be interviewed by phone or email.

At the beginning of the phone/face-to-face/electronic interviews, the researchers introduced themselves and the research objectives; the participants were asked permission to record the conversation (except for the electronic interview) and were assured that their information would remain confidential. Interviewers obtained verbal consent from the participants, and data collection started with a general question about demographic characteristics. Open-ended interactive conversation was used during in-depth interviews, and participants were encouraged to expand their views and feelings. Each interview lasted approximately 20–45 min and was recorded and transcribed verbatim. The interview guide included the following questions:

1. Tell us about your experience with COVID-19.
2. How has the coronavirus infection affected your life?
3. Do you have problems with COVID-19 condition regarding nutrition and other things?
4. What were the most critical issues you had during this period?
5. What did you or those around you do to improve your illness?
6. Could the help of others in this course be helpful to you?
7. Were you able to produce food yourself? What would you do if you could not make food?

Two researchers (a nurse and one nutritionist) conducted the interviews from July 2021 to February 2022.

#### Stage 5: data analysis, from the description of the structure of experience to its practical implications

The data analysis process was performed in three stages based on the analytic IPSE process as an inductive approach (17). This process has two stages: an independent study by individual researchers and a collective pooling of the data by the group.

##### Descriptive phase

In this individual procedure, three researchers (AHR, TKH, and SP) independently read and review the interviews several times to conduct a descriptive analysis. This process involved listening and reading the transcripts, exploring the participants' views and experiences, eliciting the descriptive units from the interviews' texts, and then regrouping these units to generate the categories based on their propinquity of meaning. During the group process phase, all group members met after every five interviews to share the categories and conduct the structuring and practical analysis phases.

##### The structuring phase

The research team consulted after every 10 interviews to fulfill the structures of experiences and categories. The categories are regrouped into carious axes of experiences so that each axis can have a meaningful relationship with their containing concepts. This stage was carried out using MAXQDA 2020 software (23).

##### Practical phase

Finally, for all group members, the practical stage of triangulation with literature leads to identifying the main aspects of the results and extracting practical concepts from them. This stage is not a process of IPSE; however, all qualitative research studies should follow it as a necessity. Finally, the literature review results were discussed among the research team members, and the similarities and differences of their results with the results of the present study were identified. The scientific article was prepared according to the consolidated criteria for reporting qualitative research (COREQ) checklist as an outline for reporting qualitative research (24).

**Figure 1:**
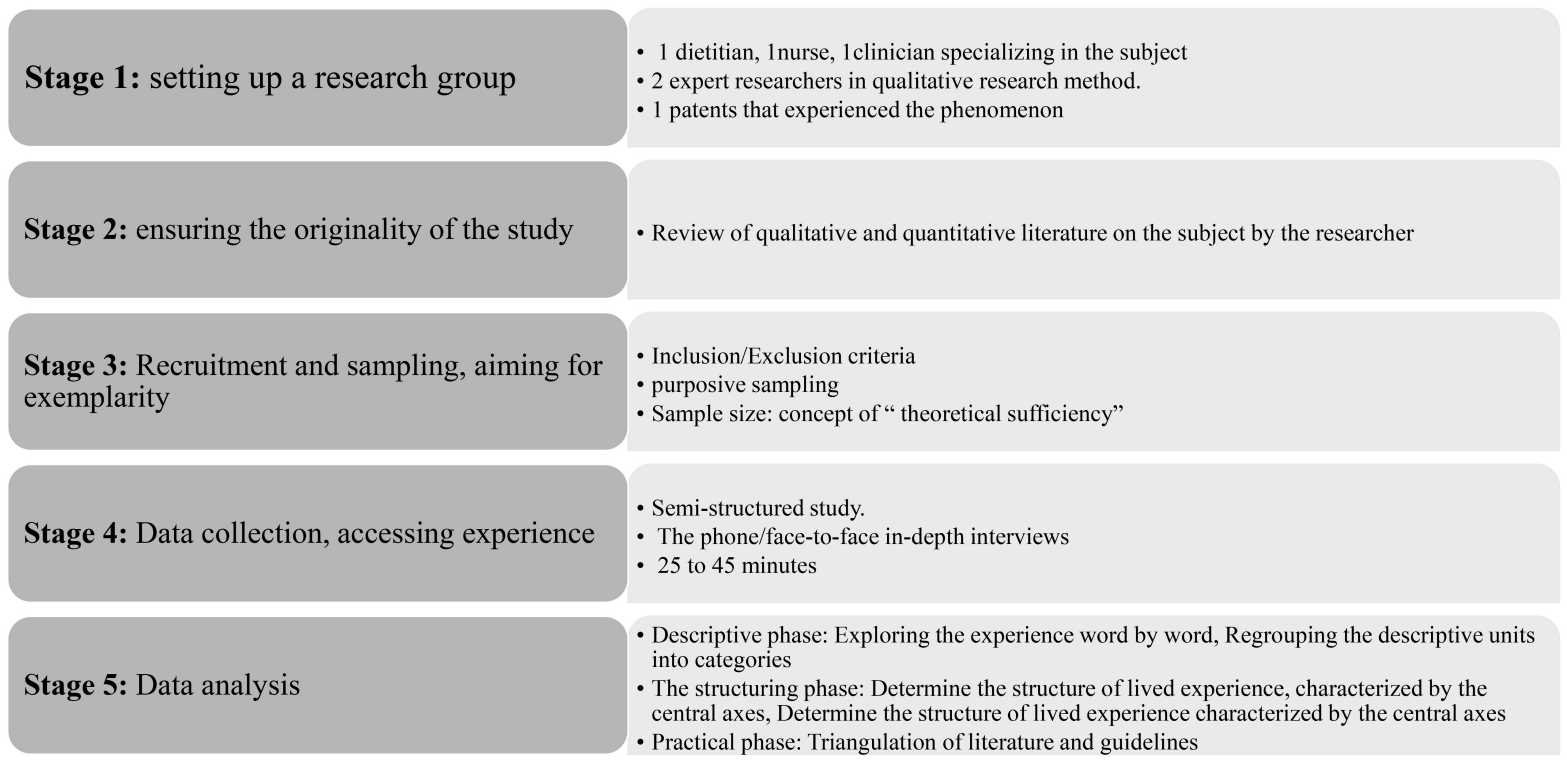
IPSE in five steps.

**Figure 2:**
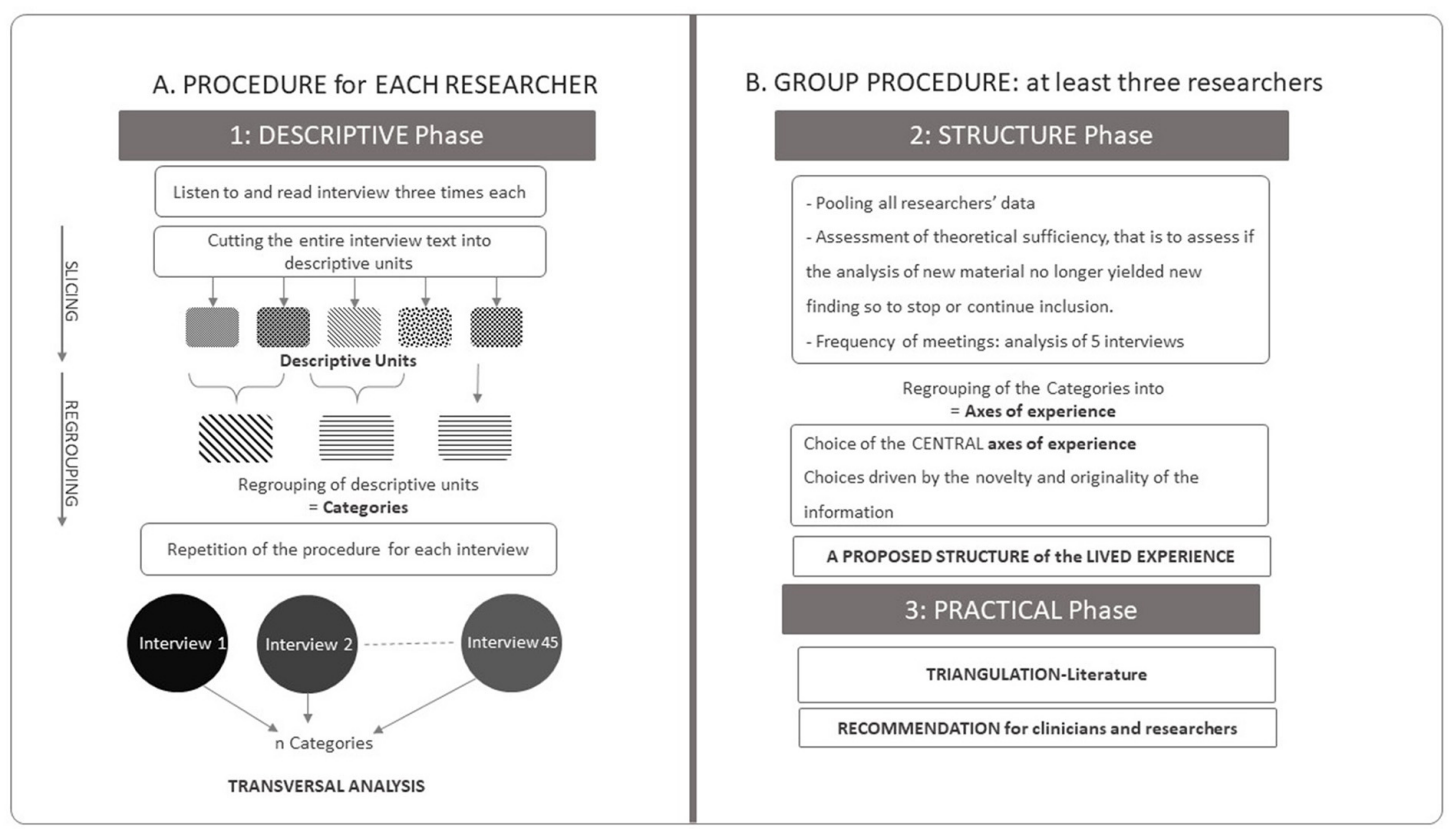
Data analysis procedure. **(A)** The procedure of each researcher, individually, corresponds to the descriptive analysis phase (including: listening to and reading the interview, cutting up the text in descriptive units and then regrouping them into categories, This operation is performed for each of the 45 interviews, which are analyzed transversally. **(B)** This structuring phase involves a group procedure (at least 3 researchers) with regular pooling of the data and analysis. During this phase, the axes of experience are produced. Finally the practical phase, which leads from triangulation by the literature to concrete proposals (“Adapted with permission from Sibeoni et al. (17), licensed under CC BY 4.0, https://doi.org/10.1186/s12874-020-01099-4”).

The summary of the analytic IPSE process is presented in Figure 1, and the data analysis procedure is depicted in Figure 2.

### Rigor and methodological quality of IPSE

Several criteria were used to ensure the quality and rigor of the current study, which was proposed for IPSE studies (17). Lincoln and Guba's criteria were followed to achieve the accuracy of the (25) results. These participants were asked to react to our proposed findings from their own experiences by sending the findings *via* email or face-to-face meetings. Data credibility was achieved by selecting participants with various treatment experiences at home or in the hospital and different severity of illness. Methodological triangulation was followed by other data collection methods such as interview, observation, and memoing, as well as investigator triangulation *via* selecting researchers with different expertise. All interviews were immediately transcribed and analyzed simultaneously—researchers' reflexivity is addressed by avoiding the effects of their assumptions when analyzing the data. Therefore, open discussion was constantly conducted between the researchers in the research group meetings. In addition, bracketing was practiced, where the researchers tried to put their previous information aside and not use it in the data analysis. A thick description was used to transform the data by explaining all stages of the study (sampling, data collection, and analysis) in detail. The inquiry audit was conducted by an experienced person other than the research team to ensure dependability. The research was conducted in Persian, the language of the researcher and the participants were in the same language, and finally, the article was written in English and sent to a professional editor.

### Ethical consideration

In this study, verbal consent was obtained from the participants, and permission to record their phone interviews was obtained from them. Also, the participants were informed that they could exit from the study whenever they wished. The assurance of confidentiality of their information was explained to the participants at the beginning of the study. This study has the ethical code IR.SBMU.NNFTRI.REC.1399.034 approved by the Research Council of the Institute of Nutrition and Food Industry Research of Iran.

## Findings

After conducting 45 semi-structured, in-depth individual interviews and reaching data saturation, the recruitment of participants was stopped. The age range of the participants was between 18 and 72 years, with an average of 39.80 ± 13.82 years. Two-thirds (*n* = 30) of the participants were treated at home and one-third (*n* = 15) of them spent part of their treatment in the hospital, of which four people were admitted to the intensive care unit. Among the participants, 10 did not take any supplements during the disease period, but the other participants received at least one vitamin C, vitamin D, zinc, and multivitamin supplements. The general characteristics of participants are shown in Table 2.

**Table 2:** Main demographic characteristics of the participants (*N* = 45).

**Variables**		**Values in number (%)**
Gender	Female	34 (75.6)
	Male	11 (24.4)
Disease history	No Disease	26 (57.8)
	Diabetes	4 (8.9)
	Obesity	4 (8.9)
	Hypertension	7 (15.6)
	Cancer	2 (4.4)
	Chronic respiratory disease	1 (2.2)
Way of infection	Family members	15 (33.3)
	Health centers	4 (8.9)
	Public places	7 (15.6)
	Workplace	7 (15.6)
	Unknown	12 (26.7)
Living situation	With family	41 (91.1)
	Alone	4 (8.9)
Help for buying or cooking foods	Yes	35 (77.8)
	No	10 (22.2)

At the same time as conducting the first interviews, the descriptive analysis phase began, and descriptive units were extracted by cutting the text up and regrouping them into categories. Data analysis explored a structure of lived experiences formed by five central axes of the experience: (1) nutritional problems; (2) physical problems; (3) psychological burden caused by the disease; (4) the supporting role of others in the disease tolerance, and (5) bitter and sweet experiences of Disease.

The relevant quotations from the interview transcripts are presented in Table 3.

**Table 3:** Quotations.

**1. Nutritional problems**	
1.1. Nausea and food intolerance	Q1: “My stomach could not hold solid food and I had severe nausea.” (F^*^, 22 yrs^**^) Q2: “I still have digestive problems and cannot tolerate foods that are high in fat or salty.” (F, 53 yrs)
1.2. Anorexia and reluctance	Q3: “It was difficult to consume solid foods, especially rice, and I did not want to eat because of the lack of smell and reduced taste.” (F, 49 yrs) Q4: “I stopped eating sweets and fatty and fried foods in general, I ate dates and honey, which are natural, but not every day, and the foods are low in oil.” (M^***^, 50 yrs)
**2. Physical problems**	
2.1. Feeling weak, tired and powerless	Q5: “It was a difficult experience and it was difficult to spend all the time in lockdown.” (M, 30 yrs) Q6: “It is a very difficult disease and it completely upsets a person in every way.” (F, 22 yrs) Q7: “I had become very weak, because I had two small children, I had to take care of their work, myself and my wife, my work had become much more, it had multiplied.” (F, 38 yrs) Q8: “My body was weak, the colorless face, and the herpes on my face was increasing.” (F, 23 yrs) Q9: “Because I have had lung problems and pulmonary fibrosis for 4 or 5 years, I saw that I was short of breath and I was doing housework before Corona, but when I got Corona, I could no longer work. Now I can only wash the dishes and if the smell of the food doesn't bother me, I cook, but I get tired quickly.” (F, 54 yrs) Q10: “A special disease that I have never experienced before. It took me a long time to fully recover and I didn't have enough energy to move for almost 2 months and I got tired very quickly.” (F, 53 yrs)
2.2. Unwanted side effects of the disease	Q11: “They treated me poorly in the ICU, I had very beautiful hair, they cut my hair, I still have hair loss, they put a very bad stitch on my chest, the last day I was discharged from the hospital, they forgot to remove my stitches, they didn't change the catheter. Because of this, I got a urinary infection, strong antibiotics were given because of that infection, and these two were the fault of the hospital, now my breasts are very ugly; I had bedsores.” (F, 36 yrs) Q12: “It was a flu that affected me for 12 days instead of 3 days! There was no other special effect and I slept at home for 12 days (M, 72 yrs)
**3. Psychological burden caused by the disease**	
3.1. Concern about the consequences of the disease	Q13: “It didn't have a special effect on my life because I was mildly infected, only at the beginning of the disease period, which of course I still didn't know that I was infected. The symptoms I had were unpleasant, for example, I had muscle pain.” (F, 20 yrs). Q14: “Well, for me, Corona was normal and the hardest thing was that my nose was blocked and I had a lot of coughs.” (M, 23 yrs) Q15: “Our appetite had increased, and this made me very nervous because I thought I would become very fat after this time.” (F, 26 yrs).
3.2. Worry about spreading the disease to others	Q16: “There is also a little concern for the danger that happens to a person, for example, my lungs do not get involved and I do not get hospitalized or I do not lose my life.” (F, 44 yrs) Q17: “It was very bad; I was very scared when I was hospitalized. I thought that I would die every moment, my child would be alone. Thank God I was safe.” (F, 67 yrs)
3.3. Loneliness, despair and feeling dependent on others	Q18: “It's as if you're trapped in a crypt where even though you hear the voices of your loved ones and know that they're near you, but you can't reach them.” (F, 49 yrs) Q19: “But I still say that I wish I didn't get the disease because I was very affected by it, I had never been hospitalized, I had never taken any pills.” (F, 36 yrs) Q20: ‘Like I said, it had a very bad effect on me mentally.” (F, 22 yrs) Q21: “One kind of doubts everything, everything and everyone around him.” (F, 47 yrs)
**4. Supporting role of others in the disease tolerance**	
4.1. Strengthening the spirit to overcome illness	Q22: “The people we love, even their voice and image, giving hope to life and their positive energy have a great impact.” (F, 40 yrs) Q23: “Their support was very good, they kept telling me that you don't have Corona... because I didn't have any of the symptoms, I was just short of breath!!!” (F, 54 yrs) Q24: “It was a very bad experience, but you realize that your family or someone you think doesn't remember you, it's not like that, they prayed for me, my brother was always with me during that time when everyone was avoiding me, my sister cooked food for me despite having an infant, and you realize how much the family loves you.” (F, 36 yrs)
4.2. Providing essential needs	Q25: “The help of others was definitely effective, if they didn't bring me food, I would have had to go out myself, which would have infected at least one or two other people.” (M, 24 yrs) Q26: “There was support from family and friends, even my grandson made a birthday cake, but due to the Corona situation, we held a private birthday.” (M, 65 yrs)
**5. Bitter and sweet experiences of disease**	
5.1. Emergence of unpleasant feelings	Q27: “At first it didn't seem to affect life that much. It affected my life and work for about a month. I had difficulty performing tasks or was unable to perform daily tasks.” (F, 47 yrs) Q28: “Fatigue and constant care for recovery held me back from many things.” (M, 23 yrs) Q29: “It was a bad experience and now if we want to go somewhere that is crowded, I tell myself that I don't want to go back to that condition and if one of the children and family members has a symptom, he wears a mask and avoids me, because I don't want to experience this disease again.” (F, 45 yrs) Q30: “It was a mysterious and painful disease that recurred and the symptoms intensified just when you felt better and the symptoms were relieved.” (F, 49 yrs) Q31: “I concluded that how close death is to a person and that a person can overcome many things that others say are terrible. I realized that one should not have stress and fear at all, because fear itself can have a much worse effect and there is nothing that cannot be overcome.” (F, 22 yrs) Q32: “It was a very bad illness, I had given birth before, and I don't think it is as severe as a COVID-19 illness.” (F, 54 yrs) Q33: “When I got sick, my children and my husband were heartbroken and worried that something would happen to me.” (F, 54 yrs). Q34: “I completely lost my sense of smell on the second day of my illness, and so did my sense of taste. When I ate, it was like I was eating paper towels.” (M, 24 yrs)
5.2. Individual and social lifestyle modification	Q35: “I had to be in quarantine, I missed my wife, children, and parents and my dog very much.” (F, 49 yrs) Q36: “No one would come near me anymore; my communication was stopped.” (M, 29 yrs) Q37: “In general, being in quarantine and not having contact with family members, as well as not doing daily tasks, at the same time as being sick, was a difficult experience I had during this disease.” (F, 49 yrs). Q38: “I feel afraid of everything around me and I try to pay more attention to hygiene.” (F, 22 yrs) Q39: “I notice more hygiene than before; we don't go anywhere except for essential work.” (M, 30 yrs) Q40: “Apart from the fact that I rested at home for 2 weeks and I was at home and I was able to catch up on my personal work, read a book, I had some ideas in my head, I thought more about them and rested more.” (M, 24 yrs) Q41: “I became a different person, my mind became more open, I saw that everyone is different, I say that one should see everything in a positive way, if someone does something bad to you, leave it to God.” (F, 45 yrs) Q42: “It was a difficult experience and it was hard to spend all the time in quarantine, but I think I miss this period because we were with my family for two whole weeks.” (M, 30 yrs)

^*^F, female.

^**^yrs, years.

^***^M, male.

**1 Nutritional problems**

This theme is detailed in another published article (26); only its most crucial subthemes are mentioned in this article (Table 3).

**1.1. Nausea and food intolerance**

Regarding nutrition during the illness, the majority of the complaints of people were about digestive problems such as nausea and intolerances, which made them unable to eat well like before the disease (Q1 and Q2).

**1.2. Anorexia and reluctance**

According to the statements of the participants, in the majority of the cases, lack of appetite and unwillingness to eat food caused food consumption to be less than people's daily needs. Due to symptoms such as pain, fever, and chills, the person's desire to consume food was reduced, and it was preferred that the consumption of food be limited to some foods that the patient likes more or does not find any problem-consuming food (Q3 and Q4).

**2. Physical problems**

**2.1. Feeling weak, tired, and powerless**

One of the most important statements of many participants was about the difficulty of the disease and the conditions that the disease created for them, and according to them, they had never felt the challenging experience of this disease before (Q5 and Q6). Some participants talked about being weak, feeling unable to do everyday tasks and responsibilities, and how the disease had prevented them from doing the simplest tasks (Q7 and Q8). Fatigue was among the physical problems that the majority of study participants mentioned as a disease complication. This fatigue limited the patient's activities during illness and continued even long after, during the recovery period (Q9 and Q10) (Table 3).

**2.2. Unwanted side effects of the disease**

Perhaps this experience was much more difficult for the patients admitted to the hospital; so, the severity of the symptoms and hardships they faced in the hospital made it harder for them to bear the disease or they faced more physical problems as one of the recovered patients who was hospitalized in the intensive care unit of the hospital expressed their experience about the disease (Q11). Some patients felt that the experience of getting infected with COVID-19 was similar to the flu or a cold, which was associated with a more extended recovery period (Q12).

**3. Psychological burden caused by the disease**

**3.1. Concern about the consequences of the disease**

According to the statements of the patients, it was concluded that in most of the cases, the mental burden caused by the disease for the person and their surroundings was high and caused even more concern for them than physical problems. This may be the difference between the consequences of this disease and other diseases. Of course, according to their statements, this effect was less common among people with mild degrees of the disease (Q13, Q14). Even one person stated that he was afraid that he would not gain weight after his illness, and this caused him emotional stress (Q15) (Table 3).

**3.2. Worry about spreading the disease to others**

The feeling of various concerns for the health of oneself and others created a significant burden in people's minds, and these multiple stresses could cause problems in proper nutrition and even the recovery period of people. The fear created in people, especially at the beginning of the epidemic, was similar to little more distant times when all kinds of infectious diseases were common in societies and killed many of the infected (Q16 and Q17).

**3.3. Loneliness, despair, and feeling dependent on others**

Being in lockdown conditions and staying away from others, feeling alone and not having a companion, and fear of getting infected again were things that the majority of people experienced with this disease (Q18–Q20). The occurrence of some negative emotions made it more difficult for people to bear the disease, such as doubting that the people around them are sick, depression, feeling bad about being hospitalized, and not having the energy to do the work that required them to get help more than before (Q21) (Table 3).

**4. Supporting the role of others in disease tolerance**

**4.1. Strengthening the spirit to overcome illness**

The majority of participants positively expressed the role of others and their help to the patients and their families. Although their physical presence was not possible due to the lockdown conditions, their distant support made it easier for them to bear the disease burden (Q22 and, Q23). The good feeling of being supported by others was a feeling that some participants were delighted and happy with, and this made them face the disease with more hope for recovery (Q24) (Table 3).

**4.2. Providing essential needs**

Due to the fact that people were not able to do some things such as shopping outside the house or cooking food during the time of infection and special conditions of lockdown, the support of people around them, both in terms of spirit or providing them the necessary foods, was considered very vital. It was possible that in case of lack of support from the surrounding people in meeting the individual's and his family's needs, they satisfied the minimum nutritional needs and experienced more weakness and misery (Q25 and Q26).

**5. Bitter and sweet experiences of disease**

**5.1. Emergence of unpleasant feelings**

According to the participants of this study, the COVID-19 disease had many bitter and sweet experiences, and duality can also be seen in these experiences at the same time; these experiences were new and challenging for some participants, but there were others for whom the opportunity of lockdown and illness was a different experience. The most unpleasant experience people expressed was falling behind in their usual life plans (Q27 and Q28).

Some patients mentioned this disease with a disturbing and mysterious interpretation, which they had not experienced in other diseases. Perhaps the application of these concepts was due to the emerging aspects of this disease, which were unpleasant and frightening (Q29 and Q30).

Several participants described the disease, which reflected the impression that was created in their minds due to the prevalence of the disease. Imagining the death of oneself and those around you was perhaps the bitterest experience of this disease for some people, which made life worthless for them. One of the participants considered this disease as a “disaster from God for humanity” (Q31). Another one of the participants made a comparison between the experience of this disease and natural childbirth to show how difficult his experience was with this disease (Q32). The mental pressure and psychological burden of the disease for some patients and their family members was such that they were under continuous stress for the possible risks of the disease for their parents or other family members (Q33). Some patients' unpleasant experiences were not having the sense of smell and taste and its prolongation in this disease, which was different from diseases such as colds and flu, in which these symptoms were experienced with a shorter duration (Q34) (Table 3).

**5.2. Individual and social lifestyle modification**

Other people's experience during this period was being forced to stay away from the people around them. This topic was complicated and unfamiliar for many participants, especially for people who had much interaction with others and had to change this behavior in the condition of illness (Q35–Q37). Despite all the bitter experiences that the participants repeatedly described in this disease, some of them looked at this disease as an opportunity for some activities and behavioral changes. For example, many people practiced hygiene more during this period, and this behavior may have been institutionalized in them over time (Q38–Q40). Changing people's attitudes toward the surrounding events and creating a positive attitude toward others was one of the results that one of the participants expressed as his experience (Q41 and Q42) (Table 3).

## Discussion

According to the result of a previous study, the infectious disease could affect dietary habits and cause several nutritional problems (27, 28). In our recent publication, the nutritional aspect of disease was discussed in detail. Still, in summary, it should be noted that our study was the first to evaluate the nutritional problem associated with COVID-19. The majority of the patients complained about gastrointestinal complications such as nausea, vomiting, solid food intolerance, and loss of appetite, which made them unable to eat as well as before the disease (26). The underlying causes of gastrointestinal problems in COVID-19 infection appear to be associated with the human host receptor ACE-2, gut microbiota, antiviral and antibiotics consumption, and inflammatory reactions that influence the digestive tract (9, 14, 29).

In the present study, the majority of the patients reported severe physical symptoms of the disease, including fatigue, fever, body tremors, myalgia, and difficulties in performing daily activities (30). However, these symptoms were reported in a more significant proportion of women. The prevalence of fatigue in patients with COVID-19 was reported to be 28–87% and continued up to 3 months post-COVID-19, and the majority of women reported it (31). In the recent randomized clinical trial, the more significant proportion of individuals reported fatigue, fever, body tremors, and myalgia in the majority of the study participants (32). In another qualitative research carried out in Iran, they revealed that patients experienced physical disorders such as fatigue (33). Moreover, the participants complained of weakness and myalgia. Myalgia is a musculoskeletal symptom characterized as pain in a muscle or group of muscles that reflects inflammation and cytokine storm in 36% of patients with COVID-19, which can continue for months, reducing the quality of life (31, 34). In addition to the musculoskeletal symptoms, COVID-19 infection has also been associated with other manifestations, including fever and body tremors. The prevalence of fever was reported at 79.43% in the meta-analysis of symptomatic adult COVID-19 patients (35). In the study by Son et al., while some patients only experienced mild myalgia, the majority of them complained of high fever and weakness (36).

Mental health is one of the most critical aspects of health. The psychological burden of disease was a neglected part of the diseases, which affects the duration of diseases and also could affect the quality of life after diseases (37).

Previous studies on the mental health impacts of COVID-19 have shown that the lived experiences of patients in affected countries indicate that awareness and preparation for infectious disease outbreaks can trigger anxiety-related mental health disturbances (38–40).

In our study, the majority of the interviewed patients were impressed and worried about their health and their family members' health. Furthermore, they reported at least one stress-related symptom, such as anxiety and depression, during their experience of COVID-19. Moreover, quarantine and being away from others, feeling alone and not having a companion, and fear of re-infection were the things that the majority of the people experienced in this disease. Previous studies indicated that COVID-19 could predispose patients and their families to anxiety and stress related to sudden uncertainty regarding the length of isolation, the risk of being infected or infecting others (41, 42).

Furthermore, psychiatric consequences of COVID-19 infection can be caused by the immune response to the virus itself which increases the production of proinflammatory cytokines (43). The elevation of these cytokines (especially IL-1β, IL-6, IL-10, IFN-γ, and TNF-α) may have a role in stress and anxiety in infected patients (44). Therefore, it is recommended that psychological support should be considered a significant issue in patients infected by COVID-19 (45). Based on our knowledge, we advocate for implementing adaptive coping skill training and disseminating relevant information during pandemics. Social networks and support, particularly from family members and close friends, may help enhance immune function, indirectly aiding the fight against the virus (46). Social support can be defined as a series of support an individual gets from their relatives, friends, and healthcare team. Psychosocial support can relieve the stress level, anxiety, depression, and insomnia (47).

In the present study, patients expressed that psychological support and help from their families had positive effects during the disease. Consistent with our findings, Yang et al. found that social support improved the psychological symptoms of patients with COVID-19 in China (46). The social consequences of COVID-19 originated in the easy transmission nature of coronavirus from one person to another, which induced obligatory isolation of patients and reduced their activity and social participation significantly (37). As we reported previously, family social support led to positive changes in dietary patterns, including alterations in the type of food and food preparation methods based on disease complications (26). Consistent with our findings, Tajbakhsh reported that the fear of virus transmission decreased the quantity and quality of communication, interaction, and family ties (48).

The COVID-19 outbreak had several sociopsychological effects worldwide, not only for infected people but also for all populations experiencing quarantine and social isolation. The bittersweet experiences of COVID-19 are another aspect evaluated in the present study. The interviewed patients expressed social isolation, missing family communication, fear of the death of themselves and family members, and loss of smell. However, some participants expressed the positive aspects of the quarantine period, for instance, having enough time to read books, do personal work, and be more conscious about hygiene and individual health. Furthermore, they had to change their attitude toward the surrounding events and create a positive attitude toward others.

In the online survey aimed to evaluate any positive effects of COVID-19 in the Dutch population, they found that 58% of participants reported positive effects of the pandemic, including rest, working from home, and feeling more socially connected (49).

### Strengths and limitations

This study was conducted to explore the nutritional experiences of COVID-19 patients in Iran. As the first study of its kind, it provides valuable insights into the multifaceted nature of these experiences. Due to the limitations of conducting interviews via voice calls, participants' non-verbal cues and body language were not observable. This may have impacted the depth and richness of the data collected. Additionally, as the participants were primarily recruited from public hospitals, the findings may have geographical limitations. Furthermore, the study's cross-sectional design limits the ability to draw causal inferences and may be subject to recall bias.

## Conclusion

Patients with COVID-19 disease experience many physical and psychosocial consequences that affect their quality of life. Therefore, sociopsychological support provided by psychologists and family members can have ameliorating effects in reducing disease consequences. Further interventional studies were needed to capture these aspects of diseases.

## Data Availability

The raw data supporting the conclusions of this article will be made available by the authors, without undue reservation.
